# School in the time of Covid

**DOI:** 10.1007/s40592-022-00161-9

**Published:** 2022-07-20

**Authors:** Shamik Dasgupta

**Affiliations:** University of California, 310 Moses Hall, 94720 Berkeley, CA USA

## Abstract

This article argues that extended school closures during the Covid-19 pandemic were a moral catastrophe. It focuses on closures in the United States of America and discusses their effect on the pandemic (or lack thereof), their harmful effects on children, and other morally relevant factors. It concludes by discussing how these closures came to pass and suggests that the root cause was structural, not individual: the relevant decision-makers were working in an institutional setting that stacked the deck heavily in favor of extended closures.

## Introduction

I will argue that extended school closures during the Covid-19 pandemic were a moral catastrophe. By this I don’t just mean that they were wrong (though they certainly were that), for that alone wouldn’t deserve mention. Stealing petty cash is wrong, but I won’t waste my time arguing the case. Rather, I mean that school closures were wrong *and* the harms they inflicted are sufficiently large that we are obliged to reflect on our mistake and ensure it never happens again. A “moral catastrophe” in this sense is therefore iterative: failing to recognize it as such invites further catastrophe down the road.

To be clear, I will not argue that it was wrong to close schools in early 2020. Brief closures of up to four weeks may have been justified and would have been consistent with pre-2020 pandemic planning recommendations from the CDC and the WHO.^1^ Rather, the closures I have in mind are those that began in early 2020 and lasted well into 2021. In the USA, these were commonplace in the west-coast states of California, Oregon, and Washington, as well as other Democratic-controlled states such as Illinois, New Jersey, and Virginia. The details vary from place to place, so I’ll use my hometown of Berkeley, California, as an example.

Public schools in the Berkeley Unified School District (BUSD) closed on March 13th, 2020. On April 6th a distance learning program began at about 20% capacity, by which I mean that students received roughly 20% of their normal instructional time (about one hour per day). This program continued until the end of the school year. Schools were still closed when the 2020-21 school year began in August, at which point the district began a new distance learning program at roughly 50% capacity (about 3 h per day). This program continued through the fall and winter. On March 29th, 2021, BUSD opened K-2 grade levels for in-person instruction at almost 100% capacity; grades 3–5 followed on April 12th. But grades 6–12 remained closed for the entire 2020-21 school year.^2^

This is an example of what I’ll call an “extended” school closure. Two notable aspects are (i) that it continued uninterrupted for over a year (roughly 1.3 years for grades 6–12), and (ii) the distance learning program involved a significant reduction in academic instructional hours (roughly 50%). Both aspects distinguish it from other school closures around the world. In many European countries, schools reopened very quickly after the first wave—Denmark reopened its schools after just 3 weeks and never closed elementary schools again.^3^ Even in the UK, where closures were used more liberally, they were targeted at surges and scattered between months of reopenings; and they typically involved a distance learning program with no reduction in academic instructional hours.^4^ Extended closures were also relative outliers within the USA, where 63% of K-12 students could attend in-person school by November 2020.^5^ Nonetheless, extended closures were widespread throughout the states mentioned above, affecting upwards of two million children in California alone.^6^

My argument that they were a moral catastrophe is directed at progressives and will draw on progressive principles. This is primarily because I believe those principles are true. But it’s also because extended school closures occurred disproportionately in progressive areas. If we’re to avoid repeating the catastrophe, it is progressives who most need to listen.

As a progressive myself, then, let me be clear that my aim is not to be backwards-looking—I have no interest in blaming or shaming those responsible for the closures. Instead, the point is to be forwards-looking and help us do better next time. For there will be a next time: another novel virus could arrive at any time, and other respiratory viruses such as influenza may surge in the wake of Covid-19. Unless we recognize these closures for the wrong that they are, we’re in danger of making the same mistake again and again. Moreover, at the end I will suggest that the root cause of the closures was structural, not individual: the relevant decision-makers were working within an institutional context that made the closures all but inevitable. So my aim is not to judge individuals but to show that institutional reform is urgently needed.

## Anredom

Pandemic mitigation is an exercise in risk management. Before evaluating school closures, it will be instructive to look at our attitude to risks associated with vaccines. The Moderna vaccine received emergency approval from the FDA in December 2020 and mass vaccinations began shortly afterwards. But the vaccine had been in clinical trials since April 2020, so in theory we could have skipped the trials and started mass vaccinations eight months earlier than we did. The delay was to test its efficacy and safety, of course, but a new vaccine is never brought to clinical trial until there is already high confidence in it (trials are expensive and involve live human subjects, after all). So, by April 2020 we already had a vaccine that was very likely to be effective and safe.^7^ Should we have skipped the trials and started mass vaccinations right away? Chappell (2021) argues we should have, thereby saving hundreds of thousands of lives lost to Covid-19 between April and December. A counterargument is that this benefit is outweighed by the small risk that the vaccine would have adverse side effects (potentially undermining trust in other vaccination programs). Personally, I don’t know what to think. It’s a difficult case.

But the following case is much clearer. Suppose, as a thought experiment, that a company named Anredom had developed a different “vaccine” by April 2020. The difference is that theirs doesn’t work, it’s just some chemical that likely has has little effect on Covid-19. Suppose moreover that the chemical can cause brain damage when injected into humans. For many, the damage is minor and and short-lived. Others experience more serious damage and require medical intervention, but they ultimately recover within a few months or a year. But for a few—say, 1%—the damage is permanent and substantial: it renders them less able to navigate life and so lowers their life-time earnings, life-expectancy, health indicators, and other quality-of-life measures. Suppose, finally, that in April 2020 we knew enough about the chemical to predict all this, both its inefficacy against Covid-19 and its harmful effects. Nonetheless, Anredom requests that we skip clinical trials and start injecting the chemical into school-aged children right away. Should we? Of course not. The idea is sickening.

*I claim that extended school closures are the same in all morally relevant respects.* In fact, the analogy would be closer if we imagine that the government *mandates* that all school-aged children must be forcibly injected with the untested chemical without consent. That would obviously be heinous—we’d have a civic obligation to protest and convince the government to change course. It would be a moral catastrophe in my sense of the term.

Extended school closures, I’ll argue, are no different. They likely had little effect on the pandemic, and this was predictable even back in April 2020 (I’ll defend this in Sects. 3 and 4). They were known to cause educational losses and mental health problems, which for some children will result in substantial decreases in quality-of-life indicators (Sect. 5). The exact risks and benefits were never tested, yet they were forced upon children without consent. In these respects (and others discussed in Sect. 6) they were just like the Anredom case.

To be explicit, my argument rests on two premises:

1. Forcibly injecting school-aged children with Anredom’s chemical in April 2020, prior to clinical trials, would be a moral catastrophe.
2. There is no morally relevant difference between that and extended school closures.

Premise (1) is undeniable and needs no further discussion. Premise (2) does all the work, so in what follows I’ll discuss a number of potential differences and argue that none are real. I cannot discuss every conceivable respect in which the cases might differ, of course, and even in the respects I do discuss some readers may insist that there are minor differences. But that’s fine—the argument goes through if I can convince you that the cases are in the same moral ball-park, so to speak, so that when evaluating extended closures we must see them through the lens of Anredom’s forced injections. That is enough to show that the closures were a moral catastrophe.

With that said, let us now turn some potential differences between the cases.

## Efficacy

The most obvious difference, you might say, is that extended school closures worked. They were effective at reducing virus transmission and thereby illness and death. Even if children were harmed, at least they (and their families and teachers) are still alive to tell the tale.

The trouble is, there’s little evidence that extended school closures worked and much evidence that they didn’t. This is an empirical matter on which there is much disagreement, but I believe the disagreement stems largely from confusions about what kind of data would settle the matter in the first place. What I’ll do here, then, is make some relatively mundane conceptual remarks about what evidence is relevant to whether extended closures worked, and the point will then be relatively clear.

So, what kind of evidence is relevant? One idea is to look at how much virus transmission occurs inside a school setting. There are numerous studies on this; most report little transmission, others report lots.^8^ But either way, this is not quite the right question. Even if in-school transmission is rampant, it could be that even more transmission occurs when schools are shut—perhaps because children are less supervised and engage in more inter-household mixing, for example. So the question is not how much transmission occurs within schools, but whether total community transmission is greater when schools are open than when they’re closed. This is a *comparative* question: we need to compare two conditions—schools open, schools closed—and ask which causes the most overall transmission.^9^ This simple point is obscured when the debate is framed as whether schools are “safe”, for that suggests that the answer can be found just by examining schools. It cannot.^10^

Moreover, what we really care about are health outcomes, not transmission *per se*. These don’t necessarily go together. For example, even if opening schools causes more community transmission, this could have little effect on health outcomes if the extra transmission is concentrated amongst those at low risk of sickness, such as children. It could even decrease negative health outcomes if households tend to do more to protect vulnerable members when their children go to school each day. So the question is really which condition—schools open, or schools closed—causes more negative health outcomes, not just transmission.

How can we answer this? The gold standard would be a randomized control trial, like we do with vaccines: you take thousands of school districts, randomly assign half of them to open and the others to close, and wait to see what happens. But this has not been done and never will be, for the simple reason that it would never be approved by an ethics review committee.

Next best is an observational study: you compare open districts with closed districts and see which tended to have better Covid-19 outcomes in the community.^11^ This kind of study is notoriously hard to do well. For one thing, you must take care not to cherry-pick. Whatever your view on school closures, you’ll always be able to find *some* district that illustrates your point. A meaningful study must include a broad, unbiased sample. And even then, there is the familiar problem that correlation does not imply causation. If you find higher transmission in open districts, that could be because schools caused it *or* because communities that opened their schools tend to be more risk-tolerant and engaged in other behavior that caused transmission. Alternatively, if you find higher transmission in closed districts, that could be because the closures caused it *or* because communities with high transmission rates were less likely to open their schools in the first place. Fortunately, statistical methods can help estimate the direction of cause and effect by controlling for confounding factors like risk-tolerance or pre-existing rates.

It’s worth noting, though, that pure correlation isn’t entirely irrelevant to causal questions. Suppose you started off *assuming* that extended closures would definitely work. And suppose you then find the CDC study by Leidman et al. 2021, which examined data on all Covid-19 cases reported in the USA between March 1 and December 12, 2020. At the end of that time-period, they report no significant difference in community case-rates between counties that had opened schools by then and those that hadn’t—if anything, open counties were associated with slightly *lower* case-rates.^12^ Unfortunately, the study didn’t try to uncover causal relationships, so we can’t conclude that extended closures had no effect on case-rates; it could be that high case-rates caused counties to keep schools closed. Nonetheless, this data should have you questioning your initial assumption—it is not longer reasonable for you to assume that extended closures definitely worked.^13^ The data is especially surprising in light of Hartney and Finger’s (2020) study into the factors that led districts to open during fall 2020. They found that local case-rates had only a “miniscule” effect; by far the strongest determiner was political persuasion, with Democratic districts significantly more likely to stay closed than Republican ones.^14^ The fact that counties that stayed closed through December 2020 nonetheless had slightly *higher* case-rates is therefore not what you’d expect if extended school closures worked.

Still, let us turn to studies designed to estimate the effect of school closures. There are numerous such studies on the initial closures during spring 2020, the results of which are mixed. Walsh et al. (2021) reviewed 32 that met certain standards and assessed each for signs of bias.^15^ Interestingly, they found that “the studies at the highest risk of bias generally reported large reductions in transmission associated with school closures, while studies at lower levels of bias reported more variable findings” (p. 23). Of the latter studies, they found that “6 out of 14 reported that school closures had no effect on transmission, 6 reported that school closures were associated with reductions in transmission, and 2 reported mixed findings” (p. 34).^16^ But regardless, these studies have little bearing on our question, for two reasons. First, school closures during spring 2020 coincided with other interventions such as business closures and stay-at-home mandates, making it difficult (in some cases impossible) to identify the effect of school closures *per se* with any confidence. And second, these studies only examine the effect of brief closures during the initial wave of the pandemic. Even if brief closures can control a surge, our question is whether extending them for a year or more in between surges did much on top of that, and these studies don’t address this.

More informative for our purposes are studies that examine what happened when schools reopened in Fall 2020. These can better estimate the effect of schools *per* se, because by that time there was greater variation in other mitigation measures such as business closures. And they bear directly on our question, for if extended closures worked it follows that opening schools in Fall 2020 would have caused an increase in Covid-19 rates. But a number of high-quality studies suggest this is not generally the case. Harris et al. (2021) examined thousands of districts across the US at the start of the 2020-21 school year to compare those that opened with those that didn’t. They found that when the local hospitalization rate prior to reopening was less than about 40 per 100,000 per week (which was 75% of all US school districts at that time), opening schools had no effect on hospitalization rates over a six-week period.^17^ A similar study by Goldharber et al. (2022) looked at districts in Michigan and Washington; they found that school reopenings in fall 2020 had no effect on community hospitalizations or deaths.^18^

Admittedly, the Goldharber study did find some evidence that reopening led to modest increases in case-rates when pre-existing rates were high.^19^ But this isn’t relevant for our purposes. Our question is what effect extended closures had over and above brief closures targeted at controlling surges, and the latter strategy differs from the former by opening schools *in between* surges. So the question is whether opening schools when rates were *not* high increased community Covid-19 rates, and the Harris and Goldharber studies suggest it doesn’t. This is evidence that extended closures had little-to-no effect.

Nonetheless, both studies are purely observational and rely on statistical methods to estimate cause and effect. Such estimates are by their nature more tentative than ones that measure the effect of an intervention (as in a randomized control trial). Two reopening studies from Germany do even better in this regard. They exploit the fact that the start and end dates of the school summer holidays vary across federal states. Moreover, the 2020 dates were fixed years earlier and so were unaffected by the course of the pandemic; nor did they correlate with other mitigation measures like business closures. This provides the opportunity for a quasi-experimental study, in which variation in holiday dates is used as a proxy intervention. Using this design, Isphording et al. (2020) and von Bismarck-Osten et al (2022) found that reopening schools after the summer break did not cause an increase in case-rates.^20^ von Bismarck-Osten et al (2022) also looked at death-rates in adults over the age of 60 and found that reopening had no effect on them either. They further report that these patterns held even in areas of high population density (and high pre-existing infection rates, though this isn’t strictly relevant as we just saw). These studies are the closest we have to randomized control trials on this topic, and therefore constitute even stronger evidence that extended school closures did little to control the pandemic.^21^

In sum, I’ve reviewed three kinds of study: (i) those on transmission within schools, (ii) those on the effect of the initial closures in spring 2020, and (iii) those on the effect of reopenings in fall 2020. I argued that only the third kind of study is relevant to our question of whether extended school closures worked, and they suggest that extended closures had little-to-no effect over and above brief closures. This is not the final word, of course, as new evidence may come to light. Still, it is enough to show that the idea under consideration—that extended school closures differ from the Anredom “vaccine” because they work—is not supported by evidence and is, as far as we currently know, most likely false.

## Knowledge

But this leads to a second potential disanalogy. In the Anredom case, we imagined that we *knew* in April 2020 that their chemical would likely be ineffective against Covid-19. By contrast, you might say, even if extended school closures were ineffective we certainly didn’t know this back then (perhaps even now we don’t know it for sure). Maybe that’s why forcibly injecting children with Anredom’s chemical is heinous while extended school closures aren’t, given what we knew back then.

Well, what *did* we know? If the suggestion is that we had *no idea* whether extended school closures would be effective, the Anredom case would indeed be disanalogous. But no matter, just modify the case. Imagine that in April 2020 we had *no idea* whether Anredom’s chemical was effective either. But hold the rest of the case fixed: imagine we *did* know it can cause brain damage and that 1% of children who receive it will suffer substantial, life-changing harms. Now, should the government forcibly inject every school-age child with this chemical without running clinical trials? Surely not! To knowingly inflict those harms on children when we *don’t know* whether it’ll do any good is only fractionally less abhorrent than knowingly inflicting those harms when we *know it won’t* do any good.

So the suggestion must be that given what we knew in April 2020, there was good reason to expect that extended school closures would be effective. Is this true? Well, who is “we”? Here we should distinguish experts from lay people. By experts I include epidemiologists and public health officials; by lay people I include ordinary citizens as well as policy makers such as state governors, school board directors, and so on. What did lay people know about the efficacy of school closures in the early days of the pandemic? Not much, presumably, but they were in a position to learn from experts. This is the *raison d’etre* of public health authorities such as the WHO and the CDC—their job is to inform policy makers.

It is therefore striking that none of their pandemic control recommendations prior to 2020 included extended school closures. The CDC’s (2007) guidance is a clear example. For a pandemic of Covid-19’s severity (category 3) their advice is to consider school closures of no longer than four weeks. Note that this isn’t “recommended”, just something to “consider” (see p. 36). They do recommend school closures for a category 5 pandemic—the highest category, which could be ten times more severe than Covid-19—but even then they advise no longer than 12 weeks. The WHO’s (2019) guidance offers similar advice, and these documents are entirely typical of their kind—at no time prior to 2020 did the CDC or WHO ever recommend extended school closures as a pandemic mitigation policy. Nor did this change during the Covid-19 pandemic. In February 2021, during the winter surge, the CDC published its “K-12 School Operational Strategy” guidelines. Even for regions with the highest levels of transmission (defined as more than 100 new cases per 100,000 people per week) they explicitly did *not* recommend school closures, just mitigation measures like masking, handwashing, and distancing where possible.^22^

It is not true, then, that lay people—including citizens and policy makers—were given any reason to think that extended school closures would be effective. To the contrary, their one source of information on the topic advised them *against* such measures before and during the pandemic. (It may be that they didn’t listen, but that’s another story; see Sect. 7.)

What about experts? Back in April 2020, did *they* have any reason to think that extended closures would be effective? Well, prior to 2020 there were no studies on school closures and Covid-19, obviously. There were studies on school closures and influenza; Jackson et al. (2013) reviewed 79 of them and the WHO (2019) updated that review with another 22 studies. Overall, they conclude that closures can have a “moderate” effect on controlling influenza—though they emphasize that the quality of evidence is “very low” and the effect size is highly variable (p. 50). Was it reasonable to assume that closures would have the same effect on Covid-19? This is questionable, but let’s suppose so.

The trouble is, all the studies on influenza examined *brief* closures of a few weeks at most, for the reason that there were no extended closures to examine! Before 2020, school closures for public health reasons were used as targeted measures to control surges and rarely lasted longer than a few weeks. Extended closures are an unprecedented intervention—prior to 2020 there were zero large-scale studies on their effectiveness on anything there were *zero* studies their effectiveness on anything. So, even if experts knew that brief closures would have the same effect on Covid-19 as influenza, that leaves open the question whether extended closures would do much more on top of that.

Here they would have been very much in the realm of speculation. Perhaps their efficacy would remain constant regardless of length. Or perhaps their efficacy would diminish over time as children find alternative ways to socialize. Perhaps those alternatives would end up *increasing* overall transmission in the long term. In April 2020, they had no way to know. But that’s all my argument needs. As we saw with the modified Anredom case, knowingly inflicting a harm on children when we *don’t know* whether it’ll do any good is still a moral catastrophe.

In fact, mundane reasoning about the dynamics of lockdowns in general suggests that the efficacy of school closures will likely diminish over time regardless of how children behave. The key point here is that the number of people who count as “essential workers” will necessarily increase over time. For a lockdown of one day, perhaps no one counts as essential: everyone could stay at home with minimal adverse effects. But if that continued for one month there’d be widespread starvation. So, a lockdown of one month must allow for the production and distribution of food. The US food industry employs approximately 12 million people. Assuming we could get by at 25% capacity for one month, that’s 3 million essential workers venturing outside their house. And that’s just food; we also need energy, water, gas, emergency health-care, fire, police, and so on. It only takes the back of a small envelope to see that a one-month lockdown must involve tens of millions of essential workers out there keeping the rest of us alive. And as they do so they will, tragically, keep spreading the virus and bringing it home to infect whoever they live with.^23^ Extend the lockdown to six months and the number of essential workers will increase further. Medical procedures that can be put off for a month can’t always wait longer; industries that can tick over at 25% capacity for one month may collapse at that level after six. Extend the lockdown to a year and there’ll be more essential workers still, all potentially spreading the virus and bringing it home to their families. Thus, as the number of essential workers increases, the marginal benefit of keeping children locked up at home will likely decrease correspondingly.

This is just *apriori* speculation, of course. But in April 2020 that is all the experts had to go on—in truth, they had no way of knowing what effect extended closures would have over and above brief, targeted ones. As time passed more evidence accumulated, but as we saw it suggested that extended closures had little-to-no effect. Thus, at no point was there good reason to think that extended closures worked.

## Safety

What about safety? We imagined that Anredom’s chemical can cause brain damage, minor and temporary in most but substantial and permanent in some. This represents the harmful effects of extended school closures on children such as educational losses and mental health problems, the severity of which also varies from child to child (and the locus of which also lies in the brain). But are these effects truly analogous?^24^

We won’t know the full effects of school closures for many years. The consequences will continue to ripple out as the affected children grow up and build their lives. Some families report that their child thrived during the closures, so we can expect some variation. And some say that “kids are resilient”, which may by and large be true. But even so, it’s undeniable that on average and overall, school closures had harmful effects on children even if the extent of the damage is not yet known.

To be clear, the claim here is not that all interruptions of school are bad. Perhaps summer vacations are essential to a child’s overall well-being. And a lucky child who misses a month of school for a family trip around the world might gain valuable and life-enriching experiences. The claim is just that *extended* school closures during Covid-19 were, on average, bad for children. Remember, extended closures involved a significant reduction in instructional hours, no access to campus, and no face-to-face contact with teachers or peers for well over a year. If you think that restricting access to education to this extent has no harmful effects, you must have a very low opinion of schools. As an intuition pump, ask yourself if you’d support continuing these cuts after the pandemic ends. I can’t imagine that anyone would.

At least, I can’t imagine that *progressives* would. Someone caught up in the moral panic about left-wing indoctrination in schools might think that reduced student-teacher contact is a good thing! But progressives are unlikely to take this attitude. Here then is one place where my argument is directed at progressives: they will surely agree that teachers add tremendous value to children’s lives, both educationally and emotionally, from which it follows that cutting student-teacher contact to this extent must be bad for children even if we don’t know exactly what and how extensive the harm is.

And that’s all my argument needs. For imagine the same is true of Anredom’s chemical: imagine we know that it causes brain damage in children, even if we don’t know exactly what and how extensive the damage is. And suppose as before that there’s little reason to think it’ll have much effect on the pandemic. Should the government mandate that every school-aged child be injected with it? Of course not; the suggestion is absurd.

Still, we can bolster the argument by estimating the impact of school closures. Let’s focus just on the education losses; set aside other effects for now. How much education did the average student lose from an extended closure? It is too simplistic just to count the number of instructional hours lost—that assumes that distance learning is as effective as in-person learning and ignores variation in circumstances (some children made up the difference with homework or private tuition, others disengaged from school altogether).

In a study for McKinsey, Dorn et al. (2020) measured the actual educational loss and found that by the end of Fall 2020, students still in distance learning programs had learned just 67% of the math they’d typically learn that calendar year.^25^ They estimated that students who remain in distance learning through the end of the school year would lose an average of five to nine months of math learning—the latter amounts to a full academic year. These losses can have snow-balling effects on future learning. After the 2005 earthquake in Pakistan, schools in parts of the country closed for 14 weeks—less than half an academic year. Andrabi et al. (2021) found that four years later, students in those areas were a full 1.5 years behind their peers in academic markers.

What effect does education loss have on long-term life outcomes? One case study is Argentina, where extensive teacher strikes occurred in varying parts of the country from 1983 to 2014. Juame and Willen (2019) found that losing one year of primary school due to strikes led to a 6.7% reduction in income once the students were 30–40 years old. That study is no outlier: Psacharopoulos and Patrinos (2018) reviewed 1120 studies across 139 countries and found that, on average, one additional year of education results in a 9% increase in earnings per year. If the focus on income feels crass, remember that income is known to determine other quality-of-life indicators such as health and life expectancy. A lost year of education therefore translates into substantial and meaningful harms on children’s lives.

To be clear, this does not amount to a “lost generation”. That hyperbolic phrase is sometimes used in the media, conjuring emotional images of an entire generation falling apart at the seams. That of course will not be the reality. But school closures don’t need to be like *that* to be seriously harmful. A 9% reduction in lifetime earnings may not pull your emotional strings, but for the affected child it will significantly impact their quality of life.

And that’s just educational losses; I’ve said nothing about the social and emotional effects on children. This is just for brevity, not because those harms are insignificant. To the contrary, once the effects on suicide rates, violent crime, drug abuse, and other predictable consequences of shutting youth out of schools for 1.3 years are accounted for, they could be far more serious than the educational losses. But more is known about the actual extent of educational loss and the long-term effects that has on children, and that’s enough to show how damaging extended closures can be.

Moreover, these statistics are just averages. I suspect many children will avoid these harms, either through luck or private tutoring and other support structures. But by the same token, other children will suffer far greater harms. They may be rare, but they are real people whose lives were upended by extended closures. Picture a high-school sophomore in February 2020 wondering whether to drop out, and the extended closure tipped the balance and he never returned. Or a 5th grader who previously excelled academically but couldn’t learn by zoom and formed a self-conception of being “stupid”, affecting her academic career thereon. Or an 8th grader who developed behavioral problems during the closure and never went to college as a result. These are the children represented in the Anredom case by the 1% for whom the chemical causes permanent and substantial harms.

Of course, no one knows whether 1% is an accurate figure. It could be less, it could be more. But my argument doesn’t depend on the exact figure. For one thing, 1% isn’t outlandish as a ball-park guestimate. Given the central role that schools play in the fabric of a community, we shouldn’t be surprised if closing them for over a year had a dramatic effect on at least *one* out of every hundred children—in fact, it would be surprising if the number was that small. And if you think that 1% is insignificant, remember that small percentages magnify when applied to large populations. If all 74 million children in the USA were subjected to extended school closures and just 1% were seriously affected, that’s 740,000 lives upended—enough to have serious macro-level effects on society.^26^ Even if I’m overestimating this by an order of magnitude, that’s still 74,000 children suffering life-changing harms. No one in their right mind would dream of forcibly injecting children with the Anredom’s chemical if it had these effects, especially if there’s little reason to think it’ll control the pandemic in the first place. Yet that’s exactly what we did with extended school closures.^27^

## Deontology

So far, I’ve argued that there’s no morally relevant difference between extended school closures and the Anredom case vis-à-vis their *consequences*: both have little effect on the pandemic and cause the same harms in children. But some think there is more to morality than consequences; considerations of rights, responsibilities, relationships, and consent also matter. This is known as “deontology”. Is there some morally relevant difference of this kind between the cases?

One deontic aspect of the Anredom case is that their “vaccine” was forced upon children without consent. No permission was sought from children or their caregivers, which is part of what’s so abhorrent about the case. But school closures were the same in this regard: children and their caregivers had no say in the matter. You might say that they were implemented by elected officials such as state governors and local school boards, who had consent by being elected. But in the Anredom case, just imagine that those same elected officials made the decision to forcibly inject children with the chemical. Regardless of whether you think elections confer consent or not, the two cases are alike in this regard.

A second deontic aspect of the Anredom case is that the intervention is “active” insofar as it involves *doing* something, namely injecting children with a chemical. By contrast, school closures are “passive” insofar as they involve *withholding* something, namely a normal education. Deontologists think that distinctions like this can be morally relevant. Killing someone yourself is different from letting them die by withholding aid, they say, even if both result in a death. Could this then be a morally relevant difference between school closures and the Anredom case?^28^

I don’t know, but if you think it is just use a different case. Imagine that in April 2020 the government proposes a ban on iron-rich foods for 1.5 years. Their idea is that an “extended iron closure” will allow workers in the meat, nut, and spinach industries to self-isolate. Now, suppose this will have little effect on the pandemic over and above brief, targeted iron closures (see Sect. 3); the main effect will instead be iron-deficiency anemia in children. This condition is known to affect neurological development and leads to decreased learning ability, which, let’s imagine, results in the same damage to children’s lives as extended school closures. An extended iron closure is obviously a terrible idea—it would be a moral catastrophe to knowingly inflict this harm on children if it does nothing to control the pandemic. And this time, the case is like school closures insofar as it involves withholding something.

A third (and related) deontic aspect of the Anredom case, you might say, is that forced injections violate a child’s right to bodily integrity—the right for children (or their caregivers) to determine what’s done to their bodies. School closures don’t infringe on this right, so is this the key difference between the cases? Well, if children have a right to bodily integrity they also have a right to education, and school closures violate the latter.^29^ So the cases are analogous insofar as both involve a rights-violation of some kind or another. Still, one might think that the right to bodily integrity is somehow more sacrosanct than the right to education, making the Anredom case a far greater wrong.

This idea cannot be dismissed lightly. It’s true that violations of bodily integrity tend to elicit particularly strong emotions. Thinking of forced injections gives me a sense of disgust—a “yuck” factor, if you will—that is less intense when I think of school or iron closures. But does this reaction reflect something morally significant, or is it just a curious feature of my psychology? We know that emotions can stand in the way of moral clarity—many people still find homosexuality disgusting, but this obviously doesn’t reflect anything immoral about homosexuality itself.^30^ Moreover, violations of bodily integrity include atrocities like rape and torture, which elicit our most visceral reactions of horror. These emotions are warranted, but we must take care not to project them onto forced injections just because they also fall under the general category of bodily integrity violations. Thus, the relevant issue is not so much the general right to bodily integrity, but the more specific right against forced injection with a small quantity of liquid. The question is whether *that* is more sacrosanct than the right to education.

Put like that, it’s difficult to see a significant difference. Think about it from the perspective of two children, one injected by Anredom and another subjected to a year-long school closure. Both children were equally harmed, let’s imagine, and both have some cause for complaint. But does the first child have *more* cause for complaint on the grounds that she would rather have suffered the harm by being shut out of school for a year? I find that hard to take seriously.

A fourth deontic aspect of the Anredom case is that it involves just one decision in April 2020, either to forcibly inject children or not. By contrast, extended school closures were the result of multiple decisions over time. Initially it was a 3-week closure to “flatten the curve”; there was no intention that they’d remain closed for well over a year. The fact that they did was the result of a sequence of decisions to remain closed a little longer, and a little longer, and so on. Is this a morally relevant difference?

I can’t see why. But for those concerned we can modify the Anredom case. Imagine that each month, the government can decide whether to forcibly inject children with another dose of the chemical or not. And imagine that what was known about the efficacy of each additional dose on the pandemic was always the same as what was known about each additional month of school closures. So (recalling from Sect. 4), during the first month of the pandemic we could reasonably expect that injecting children once would have a moderate impact in controlling the initial surge, based on past experience with influenza. But we had no idea whether continuing the injections for twelve months would do much more on top of that, and as time went on the growing body of evidence showed no clear effect of doing so. Finally, imagine that while one dose of the chemical is not very harmful, dosing children for twelve months is known to cause the kinds of harms described in the last section. Nonetheless, every month for a full year the government decides to forcibly inject children with another dose, while refusing to put this dosing regime to clinical trial. This is no more defensible than the original Anredom case and is now analogous to extended school closures in how it unfolded over time.

The fifth deontic consideration is perhaps the most interesting. It’s that even if extended school closures do little to reduce overall community transmission (Sect. 3), they may affect *who* is at risk of infection. Most obviously, they may protect teachers. The Anredom case differs in this regard: their “vaccine” does little to reduce overall transmission and has no affect on who gets infected. Could this be a morally relevant difference?

One might think so on grounds of partiality. Teachers play a central role in the lives of children and their caregivers, and often become deeply valued at a personal level. For deontologists, relationships like these can justify preferential treatment. Just think of the parent-child relationship. When I buy my daughter a birthday present, the same money could save a less fortunate child’s life. This is hard to justify on purely consequentialist grounds, but deontologists might absolve me on the grounds that *she’s my daughter*. The idea is that the parent-child relation matters, morally speaking, so that while all lives are equally valuable it’s nonetheless right *for me* to put my own child before others. Likewise, one might argue that the parent-teacher relationship matters too, in that it’s right for parents to put their child’s teacher before other members of the community. If so, a parent might reason thus: “Even if extended school closures do little to reduce overall transmission, they do protect someone who rightly matters to me, and I am happy to manage the cost to my child as best I can”.

One difficulty with this appeal to partiality is that it can cut both ways. If school closures protect teachers *and* have little effect on overall community transmission, they must put others in the community at *more* risk of infection. Who? Those who cannot maintain isolation, presumably, such as essential workers and their families. Therefore, you can justify school closures on grounds of partiality towards teachers only if you have few meaningful relationships with these other members of your community onto whom the risk will be distributed.

But the main problem is that partiality is not appropriate here. Whatever role it plays in our personal lives, it has no place in public policy. It may be right *for me* to put my child before others, but the state must remain impartial. For the state to value one group of citizens over the rest is contrary to the egalitarian principle that all people are equal. There are names for this kind of state, none of them nice. To illustrate, imagine that the Anredom “vaccine” would have little overall effect on the pandemic but will protect people with blue eyes. It would clearly be wrong for the government to mandate it, even if people you care about have blue eyes.

Can the special protection afforded to teachers be justified without partiality? Perhaps. The state also gives special protection to police officers: they are permitted to use certain kinds of force that civilians are not, which (among other things) protects them from harm while increasing the risk that they may harm civilians. If this is justifiable, it’s not (or shouldn’t be!) because the state is partial towards police. How then might it be justified?

One answer is that policing is an essential service that the state can deliver only by affording this protection to those who provide it. But if so, extended school closures cannot be justified on the same grounds. Opening schools after a brief closure does *not* threaten the state’s ability to deliver public education, as evidenced by all those regions that just used brief closures to control a surge (i.e. most of the world): education is clearly not in jeopardy there. If anything, it’s extended closures that pose a far greater threat to the state’s ability to deliver education!

Another possible justification of special protections to police is that there are certain levels of risk that no employee should be expected to take on as part of their job; without permission to use force, police officers would face unacceptable risks. But again, even if that’s right, extended closures cannot be justified on the same grounds. For when teachers work in person, numerous studies suggest that they face no greater risk from Covid-19 than the rest of the community and are therefore in no need of whatever special protections they enjoy from extended school closures. For example, Fricchione et al. (2021) examined data from the Archiocese of Chicago school system (the largest Catholic school system in the country) as they reopened in fall 2020 and found “a lower attack rate for students and school staff than for the city overall” (p. 3). And Fenton (2021) looked at data on all teachers in Scotland from March to December 2020 and found that teachers working in person were at no greater risk of hospitalization than other working-age adults.

For these reasons, it’s hard to see how extended closures can be justified by a protection they afford teachers. Still, you may be left with the nagging feeling that teachers deserve protecting. I feel this myself. But it stems, ultimately, from partiality—from the fact that they are important enough to me and my children that I value them more than others. That is all well and good in my personal life; the problem is that it is no basis for public policy. Others may feel more partiality towards police, but that obviously does nothing to justify the special protections they receive from the state.

## Comments and speculations

That completes my argument that extended school closures were a moral catastrophe. They were no better than forcibly injecting children with an untested chemical that causes brain damage and likely has little effect on the pandemic anyway.

Let me highlight four aspects of the argument. First, it makes no distinction between public and private schools. Nor does it assume that public education is an essential service. It just rests on the fact that children are harmed when access to education is restricted, which applies across the public and private sectors.

Second, I said nothing of the harmful effects of school closures on parents and teachers. This was just for brevity. Teachers reported increased stress levels during the school closures. And some parents had to leave the workforce to support children who struggled through the closures, losing benefits such as health-care. These parents were disproportionately women, making it an important topic for further study in its own right. I cannot do this here, but once all these harms are accounted the case against extended closures will only be stronger.

Third, the argument does not depend on a complex cost-benefit analysis. It would have if extended closures had substantantially reduced transmission, for in that case the costs of closures in terms of harming children (see Sect. 5) would need to be carefully weighed against the benefits of better Covid-19 outcomes. But if extended closures had little-to-no effect on Covid-19 outcomes, as argued in Sect. 3, then all we need to show is that the harms inflicted on children are significant and the cost-benefit analysis is clear.

Still, there’s a strong case that extended closures were a moral catastrophe *even if* they had reduced transmission. This time, the argument would rest on the tremendous differential effect of Covid-19 by age. Mercifully, the virus is not a serious public health threat to children. The estimated infection fatality rate for those aged 1–14 is 100 times less than those aged 35–45, *and 10,000 times less than those over the age of 85*; other health outcomes are similarly skewed. Covid-19 is a public health crisis primarily because of the threat it poses to adults and especially the elderly, not children. Thus, even if extended school closures were an effective means of controlling the pandemic (which they weren’t), they would primarily function to protect the elderly *at the expense of the young*. It is sometimes said that societies are judged by how they treat their children, and by that measure extended school closures were a disaster. In this regard, note that the traditional epidemiological justification for school closures is to protect children—that is why they were used in the past to control influenza and measles outbreaks. The Covid-19 pandemic was the first time they were used under a rationale that prioritized the elderly over the young, and I would argue that this prioritization is morally indefensible.^31^

Fourth, the argument made no mention of the differential effects of school closures across socio-economic groups. This is not because differences don’t exist, but because the argument goes through regardless. Still, the differential effects only strengthens the case against extended closures. For one thing, there is growing evidence that the children who suffered most education loss during closures are those from socio-economically disadvantaged backgrounds. Since education losses leads to financial losses later in life (Sect. 5), we can expect that extended closures will widen the income gap in the coming decades. For progressives, who place great value in social and economic equality, this is yet a further cost of extended closures. One counterargument is that socio-economically disadvantaged communities were disproportionately affected by Covid-19 and so needed the extra protection that extended closures afforded them from the virus. But as we saw, the additional protection over and above brief, targeted closures was likely minimal. Moreover, the disproportionately high case-rates in those communities were likely due to lockdown measures that allowed high-income families to stay home while low-income (“essential”) workers were out there providing for them. Having implemented such elitist lockdown measures, subjecting their children to disproportionate levels of educational losses was just more elitism still. Progressives should view this combination of pandemic control measures as a spectacular failure.

How then did extended closures come to pass? In particular, why did *progressives* disproportionately implement a policy that was so disastrous by their own lights? Here I can only speculate based on my experience in Berkeley, California. I don’t think the closures here can be explained by bad decision-making at the individual level. Rather, the relevant decision-makers were working within institutional structures that made the closures all but inevitable. I suggest that three factors were crucial here: decentralization, unionization, and political tribalism.

Start with decentralization. After the state-wide lockdown in spring 2020, California implemented a county-level reopening plan in which counties were permitted to reopen certain sectors once they met certain Covid-19 metrics. With respect to schools, the county then handed the permission down to each school district. Importantly, these were just *permissions*, not obligations: counties and school districts could choose to impose tighter restrictions. Berkeley Unified School District (BUSD) was permitted to reopen elementary schools on October 13th, 2020, and middle and high schools one month later. But because it was just a permission, it had to decide for itself whether to reopen then or stay closed for potentially the entire school year. Note that school districts in California typically operate independently of their local municipalities, so even the City of Berkeley had no authority to force the matter one way or another. BUSD was therefore left to decide the matter on its own, as were most districts around the state.

One effect of decentralization was that school reopening became decoupled from public health considerations. After all, the average school district doesn’t have their own epidemiologists to advise them. That advice would normally come from public health departments, but they had already issued their permission to reopen and the decentralized system meant there was no further role for them to play. Thus the path was paved for extended school closures that would never be recommended on public health grounds. Indeed, once it was increasingly clear how many districts were treading this path, health officials at all levels across the state issued public pleas to reopen. But by then it was too late—the decentralized process meant that reopening had long ceased to be driven by public health.

What did drive it? Here we come to the second key factor: teachers’ unions. Once a district started the school year closed, reopening would constitute a change in working conditions and required union agreement. In Berkeley, the local union is the Berkeley Federation of Teachers (BFT), which is affiliated with the American Federation of Teachers, the nation’s second-largest teachers’ union. Throughout the 2020-21 school year, BFT invented its own health metrics and reopening conditions which were far more restrictive than state guidelines, delaying elementary reopening for months and making it impossible to reopen middle and high schools at all. To take just one example, in January 2021, while the California Department of Public Health was urging districts to reopen elementary schools once their county-level case rate was less than 25 (new infections per day, per 100,000 people), BFT insisted on waiting until it was less than 4. BFT gave no evidence-based reason for this dramatic divergence from public health guidance—nor could it, since it is a small, teacher-led organization that cannot be expected to hire infectious disease experts for advice. Nonetheless, since reopening required BFT agreement, it was in a position to enforce its own health metrics and keep schools closed if it wished.

I want to emphasize that BFT and other local unions across the state did not act improperly here. Their job is to advocate for the interests of their members, and they did so effectively. It would be a dereliction of duty for union leaders to advocate for anything else. The problem was the decentralized system that gave them such control over school reopening in the first place. School closures are public health measures; yet in effect, California delegated decisions about their implementation to local union leaders with no background in public health and special interests to pursue. That said, one could argue that teachers’ unions bore some responsibility for this decentralized system in the first place. California teachers’ unions rank amongst the top five political donors in Sacramento—they even outspent oil conglomerates such as Chevron during 2020 and the first quarter of 2021. Politicians at every level seek their donations and endorsements, especially Democratic candidates. The decentralized system allowed Governor Newsom and state legislators to wash their hands of the issue and not lose favor with teachers’ unions. But however it came about, the decentralized system of leaving each district to navigate the issue on its own, together with the power that local unions had to block reopening, meant that the default was to remain closed.

One might defend decentralization on the grounds that each community should have some control over the pace of reopening, given how emotionally taxing the pandemic was on everyone. But if so, that would warrant a democratic process that involves all stakeholders including students and their caregivers, not just teachers. Thus, the problem wasn’t so much the teachers’ union *per se* (I am personally in favor of public sector unions) but the lack of a comparably powerful and well-funded organization at the bargaining table to represent the interests of students and other members of the community with a stake in public education. Either way, the unique power afforded to local teachers’ unions over the reopening process was unjustifiable.^32^

It was also terrible public health policy. Not only did it result in extended school closures (which is bad enough), it also weakened public health authorities in the long term. The point here is that restrictive public health measures are a finite resource. There is an upper limit to how long the public is willing to put up with them, so the more they’re implemented now the less appetite there’ll be for them in the future. This is why policy based on “an abundance of caution” is so dangerous: if closures one year are excessive, there may be no political will for more closures when they’re needed the year after. We saw a hint of this effect as Newsom and the state legislature forced California public schools to open fully in August 2021, during the peak of the Delta wave when Covid-19 rates were amongst the highest they’d ever been in the state. By that time, the will for closures had run dry and elected officials had no choice but to ensure schools opened come what may.^33^ Thankfully this did not lead to adverse health consequences, but what about when SARS-CoV-*3* emerges? Public health authorities are presumably aware of this issue, but there’s no reason why local school districts and union leaders should be. Delegating public health policy to the latter risks draining a finite and valuable resource; let us hope we won’t need it anytime soon.^34^

I’ve suggested that decentralization and unionization are two factors responsible for extended school closures in California. But this does not yet explain why Republican-leaning districts tended to reopen quicker than Democratic ones, despite their teachers being unionized.^35^ This brings us to the third key factor behind extended closures: political tribalism. In the face of union opposition, reopening required leadership and will from the school district. But BUSD is under the direction of its school board, which is elected by voters. So, without strong local sentiment in favor of reopening, districts were left with no incentive to reopen and union pressure would inevitably carry the day. And by summer 2020 school reopening had become deeply politicized, with the progressive left aligned firmly against it.

This should be surprising, for we’ve seen that progressive principles pointed *in favor of* reopening! Why then did school closures become a flag-ship value for the left? I cannot hope to answer this here, but presumably the politicization of Covid-19 in general played a role. Once Trump downplayed the risks of Covid-19 in February 2020, pandemic policy quickly became split down party lines. And with the election approaching in November 2020, this partisan split only deepened. So, when Trump declared in July 2020 that schools must reopen in the fall, it was perhaps inevitable that progressives would align against it. But another factor may be progressive partiality to teachers—this time not to one’s own teacher, but to the teaching profession in general. For whatever reason, progressives tend to be partial towards teachers in much the same way that conservatives are partial towards police. Faced with clear evidence of police brutality against Black men, for example, conservatives tend to align themselves behind police and the unions that protect them—hence the Blue Lives Matter movement in conservative America. Perhaps the alignment of progressives behind teachers’ unions in 2020 was a Blue Lives Matter moment for the left.

Regardless of why the issue politicized in the direction it did, the politicization became extreme. It wasn’t just that you could predict someone’s view on the issue by their political group; it was that one’s view on this issue became a condition of group identity. This is what I mean by political *tribalism* (as opposed to mere polarization).^36^ The cost to progressives of expressing a preference for reopening was therefore high—they faced fierce social condemnation from their in-group, including accusations of racism and white supremacy. For example, in December 2020, the Chicago Teachers’ Union tweeted that “The push to reopen schools is rooted in sexism, racism, and misogyny.” And in March 2021, Cecily Myart-Cruz, head of United Teachers of Los Angeles (the city’s largest teachers’ union) described school reopening as “a recipe for propogating structural racism”.^37^ And as late as May 1, 2021, the *New York Times* reported on protesters describing school reopening as “white supremacy at its best”.^38^

Such was the climate in which school reopening was discussed. One obvious implication is that progressives who favored reopening were incentivized to keep their opinion private. No surprise, then, that elected school boards in progressive districts heard little incentive from voters to reopen.

In fact, the effect of tribalism was likely stronger than this. In a tribal climate, voicing an opinion functions not so much to inform others what you think as to express allegiance to your group. Those with no firm opinion are therefore incentivized to toe the party line when asked. And given the high cost of being misunderstood as being “on the other side”, even those who agree with the group have an incentive to *voice* an opinion a little more extreme than the group’s average. The dynamics are then obvious: voiced opinion will become a bit more extreme over time, on average, generating an incentive to voice an opinion that’s a bit more extreme than *that*, and so on. This predicts that elected school boards in progressive districts would perceive even less electoral incentive to reopen than suggested above, and indeed strong electoral pressure to stay closed.^39^

This then is my speculative account of how extended school closures in California came to pass. Decentralization meant that the issue was decoupled from public health considerations and driven by local politics instead. The unique power that local teachers’ unions held over the process meant that the default would be to remain closed. And political tribalism meant that elected school boards in progressive districts had little counter-balancing incentive to reopen.

Note that I’ve said nothing of the incentive that decision-makers have to avoid visible and punishable mistakes.^40^ To use a famous example of Milton Friedman’s, when FDA officials decide whether to approve a drug there are two kinds of mistakes they can make. They might approve a drug that causes harm, or they might *not* approve a drug that *would have* brought relief. Even if both mistakes are of equal magnitude, they have more incentive to avoid to the former since it is more visible and hence more likely to result in punishment (such as public criticism or loss of employment). Likewise, when deciding when to open schools one might make the mistake of opening too soon and causing infection, or opening too late and harming children in ways that may take years to become apparent. Again, even if the both mistakes are of equal magnitude, the former is more immediately visible; hence (the idea is) decision-makers had an incentive to err on the side of longer closures. This factor may have had some effect, but it doesn’t explain *extended* closures because decision-makers in places such as Europe presumably faced similar incentives but didn’t close schools for over a year. What I’ve tried to do is identify factors unique to California, at least, that might explain why its school policy was so different. The three factors I identified—decentralization, unionization, and tribalism—predict California’s policy without appeal to this Friedman-esque effect, for they imply that school boards in progressive districts had little incentive to open *even if* both mistakes were equally likely to be punished. Still, if the Friedman-esque effect was in play that would obviously decrease their incentive to open even further. There may even be some interplay between these factors, for the tribal dynamics above would likely magnify the Friedman-esque effect by making the first kind of mistake (of opening too early) even more salient.

If my account is accurate, it suggests how we might do better next time. First, be aware that decentralization can decoupling pandemic mitigation policy from public health, allowing it to be driven by local politics instead. As well as leading to bad policy in the short term, this also risks weakening public health authorities in the long run as mentioned above. Thus, decentralization should either be avoided, or measures should be taken to minimize these risks such as providing local decision-makers with public health advisors. Second, whether decentralized or not, ensure that the interests of all parties are democratically represented in the decision procedure, not just the interests of the biggest political donor. Special interests cannot be expected to dictate good pandemic policy. Finally, avoid political tribalism if at all possible. This is easier said than done, to put it mildly, and we cannot assume it will be addressed before the next pandemic hits (though even during Covid-19, leaders at all levels could have publicly condemned the inflammatory rhetoric about white supremacy and the like). But the first two recommendations are eminently actionable—indeed, countless states and nations around the world had the good sense to follow them throughout the Covid-19 pandemic. I leave it to policy professionals to determine what reforms are needed to implement them in California.^41^

## Footnotes

1 See for example CDC (2007, p. 12) and WHO (2019, pp. 48–53).

2 From late April, students in grades 6–12 were invited into schools for 3–4 h per week, but this time was used for “enrichment activities” and not curricula instruction. I will categorize this as “closed”. One could use the term differently, but note that my decision to talk this way only makes my life harder: it means I must argue that closures were a moral catastrophe *even if* they included limited in-school time of this kind.

3 Data from UNESCO; see https://en.unesco.org/covid19/educationresponse#durationschoolclosures.

4 The exception was the initial closures in spring 2020, when distance learning programs were more varied across the country.

5 Burbio.com tracked school reopening data for the 2020-21 school year; see https://cai.burbio.com/school-opening-tracker/.

6 This estimate is from Burbio.com. More than a quarter of a *billion* children in India were affected by extended closures, many of whom had little-to-no access to virtual instruction. That deserves its own discussion given the distinctive socio-economic conditions there; here I focus on extended closures within the US.

7 Remarkably, the vaccine was already designed in January 2020, just two days after the genetic sequence of the virus had been released! I’m using April 2020 as a (conservative) estimate of a date by which we had confidence in its efficacy and safety, as evidenced by the fact that FDA-approved clinical trials began then. Details of the process by which the FDA approves clinical trials can be found on their website; see for example https://www.fda.gov/vaccines-blood-biologics/development-approval-process-cber/vaccine-development-101.

8 The well-publicized studies from North Carolina (Zimmerman et al. 2021) and Wisconsin (Falk et al. 2021) reported very low levels of in-school transmission. Similarly low levels were reported from Australia (McCartney 2020), France (Fontanet 2020), Ireland (Heavey 2020), Switzerland (Ulyte et al. 2020), Florida (Doyle et al. 2021), and England (Ladhani et al. 2021). Higher levels of in-school transmission were reported from Israel (Stein-Zamir et al. 2020) and Finland (Dub et al., preprint).

9 Harvard and Winsberg (2021) develop this point more generally with regard to Covid-19 policy. They distinguish two senses in which an intervention “causes” an effect: a mechanistic sense on which a process leads from the intervention to an effect (e.g. a transmission event in school produces positive cases), and a counterfactual sense on which the intervention results in more of the effect than there would have been otherwise (e.g. more positive cases when schools are open than closed). They argue that evaluating Covid-19 policies requires focusing on the latter sense. I’m making the same point in the context of schools.

10 Another misfortune of framing the debate as whether schools are “safe” is that it suggests that the answer depends on one’s subjective level of risk-tolerance. What I consider safe you may consider dangerous, not because we disagree on the risk but because I’m happy to take risks you are not. The comparative question contains no such subjective element.

11 This could involve looking at the same district over time.

12 Specifically, “as of the week beginning December 6, aggregate COVID-19 incidence among the general population in counties where K-12 schools offer in-person education (401.2 per 100,000) was similar to that in counties offering only virtual/online education (418.2 per 100,000)” (p. 90).

13 In Bayesian terms, let H be the hypothesis that extended closures worked and let E be the CDC data. Then the point is that P(H | E) is not very high, so if you started off with a high prior credence in H then upon discovering E you should decrease your credence in H.

14 The study is based on data from over 10,000 (more than 75%) of the nation’s public school districts, and finds that political persuasion is the dominant factor even when controlling for factors such as health conditions, district demographics, the range of resources available to Republican vs. Democratic-leaning districts, and state-level political and economic differences.

15 “Bias” here includes cherry-picking, failing to control for potentially spurious factors, as well as other mistakes that reduce the evidential value of the study.

16 Four studies were considered to have particularly low levels of bias: Courtemanche (2020), H﻿siang﻿ (2020), Li (2021), and Matzinger (preprint). The first two found no effect, the latter two found reduced transmission.

17 They control for a range of factors such as Covid-19 rates prior to opening, local political persuasion, whether venues like bars and restaurants were open, and others besides. It also uses teacher bargaining power as an instrumental variable to further tease out causal relationships.

18 Their data on hospitalizations was restricted to Washington; their data on deaths was drawn from both states.

19 This could have been due to increased testing once schools opened; unfortunately the study did not evaluate that possibility. However, Courtemanche et al. (working paper) looked specifically at reopenings in areas of high pre-existing case-rates while controlling for testing rates, and found evidence that reopenings can indeed cause increased transmission in those conditions. In both studies, “high” corresponds roughly to more than 100 cases per week per 100 K population (the CDC’s highest category in their 2020-21 guide to reopening schools).

20 In fact, case-rates decreased when schools reopen, but von Bismarck-Osten et al. attribute this to the fact that testing and case-rates increased during the two weeks prior to reopening due to children being tested before school started.

21 Note that schools in Germany used fairly rudimentary mitigation strategies during fall 2020. There were no social distancing or masking requirements, just guidelines on basic hygiene practices, isolating symptomatic students, and ventilating when possible. (The only exceptions were two federal states that used masking for 2–3 weeks after reopening.) This means that evidence from these studies cannot be chalked up to school conditions unique to Germany.

22 Did districts that remained closed do so because they couldn’t provide these measures? Perhaps in some cases, but certainly not all. BUSD was equipped to provide them in fall 2020 but remained closed for another six months.

23 Hence the uncomfortable truth that progressives like to ignore, that the lockdowns they celebrated in the face of conservative opposition had the effect of concentrating viral transmission within working class communities.

24 In this section I compare only the *effects* on a child’s life of Anredom’s chemical vs. school closures. One might think that even if the effects are equal, causing it via brain damage vs. via school closures is a morally relevant difference. I’ll discuss this in Sect. 6.

25 This is based on data on over 350,000 students across 25 states, comparing their growth from fall 2019 to fall 2020 with average fall-to-fall growth over the three previous years.

26 For comparison, Levitt and Dubner (2005) famously argued that the spike in abortions following Roe vs. Wade in 1973 helps explain the dramatic decrease in national crime figures in the early 1990s. Nationwide, that spike consisted of 900,000 abortions in the three years following 1973. If 900,000 additional abortions had significant macro-level effects, we can expect that 740,000 severely harmed children would too.

27 You might object that the cases are disanalogous because the harms of school closures are more easily reversible than the harms of a medical intervention. School districts could start programs aimed at supporting particularly hard-hit children, for example. This may be true in theory, but the reality is far murkier. It assumes enough public will to fund such measures, and even well-funded educational interventions tend to have modest effects at the population level. Moreover, it will be even more challenging to aid those who dropped out of the school system altogether (and who may be amongst those most harmed). Clearly, the harms of school closures are *at best* only partially reversible. So, imagine that the same goes in the Anredom case: imagine that with enough public will and funding, *some* of the harm in *some* children could be reversed. Clearly, this does not change the moral repugnancy of the case.

28 This would not quite be the distinction between killing vs. letting die gestured at in the text, because letting die typically involves having no causal influence on the process that led to their death. With school closures, by contrast, the relevant decision-maker closed them and so had a causal influence. Still, the suggestion is that a related distinction between doing vs. withholding might be what makes the difference between the cases.

29 See article 26 of United Nation’s Universal Declaration of Human Rights, which states that “Everyone has the right to education”. I should say that I am skeptical that the framework of rights is a helpful way to illuminate moral problems. In my opinion, talk of rights is a shorthand way of indicating that there are moral considerations in the vicinity, and we would do better to discuss those considerations directly. But I will bracket these concerns and engage with the current idea on its own terms.

30 For a review of literature on this topic unfortunate phenomenon, see Wang et al. (2019).

31 Indeed, one could argue that prioritizing the elderly over the young is contrary to *progressive* values in particular. Winsberg (2020) does this by comparing Covid-19 to climate change. He argues that the real issue about climate change policy concerns *not* whether there is anthropogenic climate change (of course there is) but our temporal discount rate; that is, how much value we place on the welfare of people in the future suffering the effects of climate change, vs. the value we place on the welfare of people today who must sacrifice to mitigate those effects. The progressive attitude is that the discount rate should be low; that is, future welfare matters (almost) as much as welfare today. This is what justifies substantial climate change policies that require us to sacrifice today so that the next generation may enjoy a decent life in the future. It is hard to see how this attitude is consistent with harming children to help the elderly.

32 Indeed, I would argue that local union leaders have a legitimate complaint against the state for placing this burden of moral responsibility onto them when they have no qualifications for the task.

33 Note that Newsom was facing a recall election in September 2021. Political commentators speculate that the recall effort succeeded in forcing the election largely because of frustration with restrictions such as school closures.

34 In fact, this is a further analogy between extended school closures and Anredom’s “vaccine”. The reason we subject a new vaccine to stringent clinical tests is not just to ensure the safety of that particular product; it’s also to preserve trust in vaccination programs more generally. Unintended adverse affects from Covid-19 vaccines could wreak havoc on, say, measles vaccination rates. We would *never* delegate vaccine approval to, say, a local third-grade teacher and a local school board with no training in public health. But that’s exactly what California did with school closures.

35 See Hartney and Finger (2022) for data on the extent to which the length of school closures depended on local political persuasion.

36 More fully, social scientists distinguish three kinds of political polarization. “Issue-position polarization” is the extent to which policy preferences on a particular issue reflect extreme rather than moderate positions; “ideological consistency” is the extent to which (for example) Americans express uniformly liberal or conservative opinions across a range of topics; and “social polarization” (or “affective polarization”) is the extent to which people’s sense of identity becomes associated with their political group (membership of which requires ideological consistency). What I’m calling “tribalism” corresponds to this last notion.

37 See the *Los Angeles Magazine*, August 26, 2021, “Cecily Myart-Cruz’s Hostile Takeover of L.A. Public Schools”.

38 The article was titled “Restaurants and Broadway Are Coming Back. What About Our Schools?”.

39 High-quality polling would help mitigate this effect, of course, but the average local school district is in no position to employ professional pollsters; this is another problem with decentralization. Note that a similar dynamics might also explain the extent of the politicization to begin with. Theoretically, all it takes is two groups that start with *slightly* diverging opinions on an issue tied to group identity. The above dynamics then predicts that *voiced* opinion will diverge over time; insofar as one’s actual opinion is shaped by what one hears, the result is that *actual* opinion will diverge over time too. This is a highly idealized model, of course, and in the real world many other factors are in play. But it may have some effect.

40 Thanks to Peter Godfrey-Smith for correspondence on this point.

41 This article was first drafted in June 2021, just after extended closures came to an end. I would like to thank Thomas Abt, Francois Balloux, Stefan Baral, Richard Chappell, Zubeida Dasgupta, Monica Gandhi, Peter Godfrey-Smith, Hrishikesh Joshi, Sudha Laxshmi, Brandon Warmke, and Eric Winsberg for extremely helpful feedback.
